# Endothelial proteomic response to *Dirofilaria repens* somatic antigens and the Dr20/22 protein reveals parasite-driven modulation of angiogenesis

**DOI:** 10.1186/s13071-026-07314-3

**Published:** 2026-08-20

**Authors:** Manuel Collado-Cuadrado, Ana Montero-Calle, Sara Vázquez-Ávila, Rodrigo Barderas, Mateusz Pękacz, Anna Zawistowska-Deniziak, Javier Sotillo, Miguel Pericacho, Rodrigo Morchón

**Affiliations:** 1 Zoonotic Diseases and One Health Group, Faculty of Pharmacy, Centre for Environmental Studies and Rural Dynamization (CEADIR), University of Salamanca, 37007 Salamanca, Spain; 2 Chronic Disease Programme, UFIEC, Instituto de Salud Carlos III, Majadahonda, 28220 Madrid, Spain; 3 Proteomics Core UCCTs, Instituto de Salud Carlos III, Madrid, Spain; 4 Reference and Research Laboratory in Parasitology, Centro Nacional de Microbiología, Instituto de Salud Carlos III, Madrid, Spain; 5 CIBER of Frailty and Healthy Aging (CIBERFES), 28029 Madrid, Spain; 6 Pathogen Immunobiology Group, Institute of Experimental Zoology, Faculty of Biology, University of Warsaw, 02-096 Warsaw, Poland; 7 Department of Physiology and Pharmacology, University of Salamanca, 37007 Salamanca, Spain; 8 Biomedical Research Institute of Salamanca (IBSAL), University of Salamanca, Salamanca, Spain

**Keywords:** Somatic antigen, Dr20/22, Dirofilaria repens, Endothelial cells, Proteomic, Angiogenic process

## Abstract

**Background:**

Although *Dirofilaria repens* induces perivascular proliferation in subcutaneous nodules, the molecular factors driving this angiogenic response remain unknown. The objective of this study is to investigate the activation of the angiogenic mechanism in an in vitro model of human vascular endothelial cells (HUVECs) stimulated with *D. repens* somatic antigen (DrSA) and Dr20/22 protein, identifying the key pathways underlying the vascular pathology caused by the parasite.

**Methods:**

After 24 h of stimulation with vascular endothelial growth factor A (VEGF-A), DrSA, Dr20/22, DrSA+VEGF-A, and Dr20/22+VEGF-A, cell viability was assessed, and proteins from the cell lysate and supernatant of HUVEC cultures were processed. Proteomic profiles were analysed by data-independent acquisition/label-free quantitation/liquid chromatography–tandem mass spectrometry (DIA-LFQ LC–MS/MS), and bioinformatics analyses (Gene Ontology, enrichment pathway analysis) were performed to identify proteins differentially expressed in relation to the angiogenic process in subcutaneous dirofilariasis.

**Results:**

Supernatant analysis showed that DrSA+VEGF-A and Dr20/22+VEGF-A induced the most robust pro-angiogenic response compared to single treatments. Co-stimulation significantly upregulated key secreted proteins, including angiopoietin-2 (ANGPT2), cadherin-5 (CDH5), and multimerin-1 (MMRN1). This synergistic secretome, characterized by enriched angiogenesis pathways, identifies secreted endothelial cell-specific molecule 1 (ESM1) and matrix metalloproteinase-2 (MMP2) as primary drivers of vascular activation and remodelling in the host–parasite interface.

**Conclusions:**

DrSA and Dr20/22 act as key molecular drivers that manipulate host angiogenesis. While their synergy with VEGF-A clarifies the origin of vascular changes in subcutaneous dirofilariasis, further research is essential to fully characterize the specific pathways governing vascular pathology and subcutaneous nodule formation. These investigations will deepen the scientific understanding of host–parasite interactions and the disease's clinical etiopathogenesis.

**Graphical Abstract:**

**Figure:**
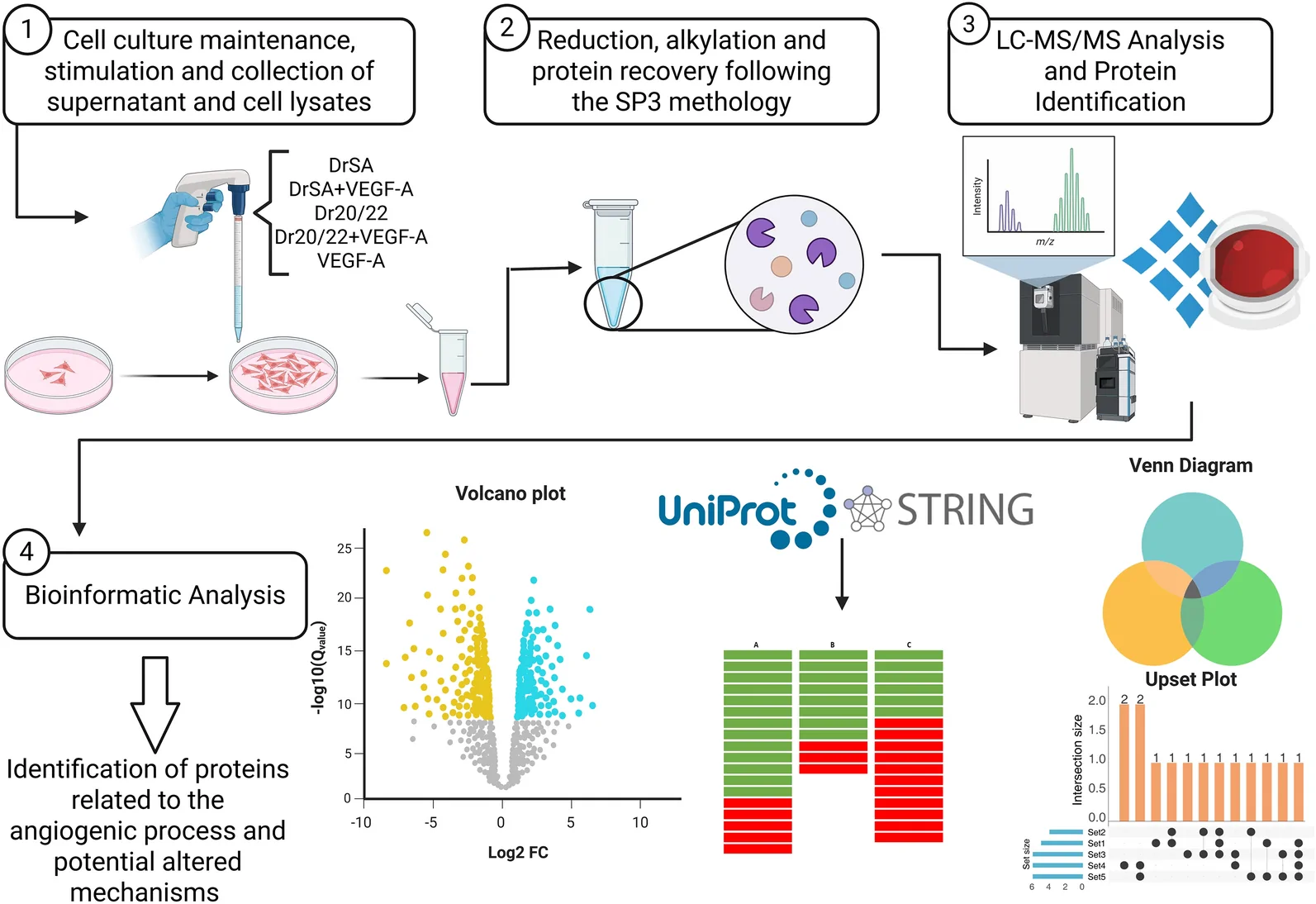

**Supplementary Information:**

The online version contains supplementary material available at 10.1186/s13071-026-07314-3.

## Background

Angiogenesis is a dynamic and tightly regulated process by which new blood vessels form from the existing vasculature. It is essential for embryonic development, wound healing, and tissue regeneration, but also occurs under pathological conditions such as chronic inflammation, tumour progression, and parasitic infections [1, 2]. Pro-angiogenic signalling is primarily mediated by vascular endothelial growth factor (VEGF)-A binding to its receptor vascular endothelial growth factor receptor 2 (VEGFR-2), which activates downstream cascades involving phosphatidylinositol-3 kinase (PI3K)/protein kinase B (AKT) and mitogen-activated protein kinase (MAPK) pathways that promote endothelial proliferation, migration, and survival [3]. Other mediators, including fibroblast growth factors (FGF), angiopoietins, and matrix metalloproteinases, facilitate extracellular matrix degradation and capillary morphogenesis [4]. In contrast, antiangiogenic pathways are triggered by inhibitory factors such as thrombospondin-1, endostatin, and angiostatin, which block VEGFR-2 phosphorylation or induce endothelial apoptosis, maintaining vascular quiescence [5, 6]. The dynamic balance between these two signalling arms determines the initiation, maintenance, or inhibition of angiogenesis.

*Dirofilaria repens* (Railliet & Henry, 1911) is a mosquito-borne zoonotic filarial nematode widely distributed in Europe, Asia, and Africa [7, 8]. Dogs act as the main definitive host and reservoir, harbouring adult worms in subcutaneous tissues, where they release microfilariae into the bloodstream. These are taken up by mosquitoes of the genera *Aedes*, *Culex*, or *Anopheles*, where they develop into infective third-stage larvae (L_3_) that are transmitted to new hosts during feeding. In humans, who serve as accidental hosts, immature adults can cause subcutaneous or ocular nodules often characterized by local inflammation and perivascular proliferation [9, 10]. These findings indicate that vascular and immune components are closely involved in the host’s response to *D. repens*.

Angiogenic processes have been described in a variety of parasitic infections as a host–parasite interface that contributes to parasite survival or tissue repair. *Plasmodium falciparum* induces endothelial activation and microvascular dysfunction during cerebral malaria [11], while *Leishmania infantum* promotes VEGF expression and matrix remodelling in infected tissues [12]. Likewise, *Schistosoma mansoni* eggs secrete proteins that stimulate perigranulomatous neovascularization [13]. In helminth infections, *Echinococcus granulosus* and *Trichinella spiralis* promote local capillary proliferation as part of tissue adaptation [14, 15]. In *D. immitis*, excretory/secretory antigens activate host plasminogen, degrade extracellular matrix components, and stimulate endothelial migration [16]. In contrast, somatic antigens exhibit modulatory or even inhibitory effects on capillary-like structure formation, suggesting a more complex regulation of angiogenesis [17, 18].

Although *D. repens* has been associated with perilesional vascularization in both canine and human infections [8], the molecular mechanisms underlying the endothelial responses upon parasite infection remain poorly characterized. Recent in vitro studies have shown that *D. repens* somatic antigens (DrSA) modulate the expression of pro-angiogenic mediators such as VEGF-A and endoglin, altering endothelial morphology and migration [19–21]. To further explore these effects, we analysed parasite-derived preparations, including DrSA from adult *D. repens* as well as individual larval-related molecules. One such molecule, Dr20/22, is an L_3_ antigen associated with early dog–parasite interaction. Although its biological function remains unknown, its association with early tissue invasion suggests a potential role in modulating endothelial or immune cell responses [22]. Its potential involvement in angiogenesis regulation has not been addressed, but its presence in endothelial stimulation assays highlights it as a candidate molecule capable of influencing vascular activation or inhibition.

Therefore, the objective of the present study was to analyse the effect of the DrSA and the recombinant protein Dr20/22 on the angiogenic process in a vascular endothelial cell model through a comparative proteomic study to identify differentially regulated proteins and molecular pathways and provide information on their contribution to the vascular pathology observed in subcutaneous dirofilariasis.

## Methods

### Antigen preparation

Adult DrSA were prepared as described previously, with some modifications [20]. In brief, three adult *D. repens* worms extracted from a human subcutaneous nodule were washed in 1× phosphate-buffered saline (PBS), macerated at 4 °C, and sonicated in PBS pH 7.2 with five cycles of 70 kHz (30 s each cycle). The homogenate was centrifuged at 16,000×*g* for 30 min and the supernatant was dialysed against 0.01 M PBS, pH 7.2. The final concentration was quantified using a commercial detergent-compatible (DC) protein kit (Pierce).

The Dr20/22 protein was prepared following the methodology described by Pękacz et al. [22]. In summary, complementary DNA (cDNA) was generated from RNA extracted from adult *D. repens* worms containing the sequence of interest and inserted into the pPICZα A expression vector. The vector containing the sequence of interest was used for electrotransformation of the yeast strain *Pichia pastoris* X33. Polymerase chain reaction (PCR)-positive colonies were used for protein expression in buffered methanol complex medium (BMMY) containing 0.5% methanol at 29.8 °C for 72 h. Protein purification was performed using the Protino Ni–NTA Agarose kit for the purification of HIis-tagged proteins (MACHEREY-NAGEL). The concentration of the purified Dr20/22 protein was determined using the BCA (bicinchoninic acid) Protein Assay Kit (Pierce).

### Cell culture, stimulation, and protein sample processing for mass spectrometry

Human umbilical vein endothelial cells (HUVECs) were used in this study as an in vitro model and were maintained under controlled conditions (37 °C, 95% humidity, and 5% CO₂). HUVECs came from the umbilical cord of a healthy baby. Prior to cell culture, the surface of the plates was incubated with a matrix containing 0.1% porcine gelatin (Sigma Chemical Co., St. Louis, MO, USA), 0.01% fibronectin (Sigma-Aldrich, St. Louis, MO, USA), and 0.01% collagen (Corning, NY, USA). HUVECs were grown in Basal Endothelial Medium 2 (Lonza, Walkersville, MD, USA) supplemented with SingleQuots (Lonza, Walkersville, MD, USA). The culture medium was changed every 72 h. Cell expansion was performed by trypsinization (trypsin/ethylenediaminetetraacetic acid [EDTA], Lonza, USA) upon reaching confluence of approximately 95% to expand the cell culture and carry out the relevant treatments.

HUVECs were treated in 60-mm plates with 1 μg/μl of VEGF-A, DrSA, DrSA+VEGF-A, Dr20/22, and Dr20/22+VEGF-A for 24 h, and both the culture supernatants and cell lysates were collected and stored at −80 °C until further analysis. Untreated cell cultures were used as controls. All treatments were performed in triplicate. Protein quantification of each sample was analysed using a commercial BCA protein quantification kit (Pierce™ BCA Protein Assay Kits). Twenty micrograms of each sample was then resuspended in 100 µl of RIPA (radioimmunoprecipitation assay) buffer. Subsequently, disulphide bonds were reduced by adding 11 µl of 100 mM tris(2-carboxyethyl)phosphine (TCEP, PanReac AppliChem, Barcelona, Spain), followed by incubation with shaking for 45 min at 37 °C. Subsequent alkylation of the proteins was performed by adding 12 µl of 400 mM chloroacetamide (PanReac, AppliChem) followed by incubation for 30 min at 600 rpm in the dark.

Protein immobilization was performed using a methodology based on SeraMag magnetic beads, previously optimized [23]. Basically, 100 µl of the beads (50% hydrophilic; 50% hydrophobic) and 200 µl of 100% acetonitrile (PanReac AppliChem) were added and incubated with constant agitation for 35 min at room temperature. Once the beads were separated using a magnetic support, the supernatant was removed and the sediment was washed twice with 200 µl of 70% ethanol and once with 200 µl of acetonitrile, followed by brief air drying. The beads with the immobilized proteins were resuspended in 100 µl of a digestion buffer containing 20 µg of trypsin in 2 ml of 200 mM ammonium bicarbonate (pH 8.0; PanReac AppliChem) for 14 h at 37 °C with agitation. After incubation, for protein recovery, the samples were sonicated, vacuum-dried, and stored at −20 °C until LC–MS/MS analysis.

### Cellular viability and cytotoxicity assays

Subsequently, cell counts were performed using the Countess^®^ Automated Cell Counter (Invitrogen) following the manufacturer’s instructions to analyse the cellular viability (living and dead cells after treatment). Cytotoxicity was assessed in the supernatant of the treated and control cell cultures using the ToxiLight BioAssay Kit (Cambrex, Verviers, Belgium) following commercial instructions. This commercial kit quantitatively measures the release of adenylate kinase from damaged cells.

### Data-independent acquisition/label-free quantitation/liquid chromatography–tandem mass spectrometry (DIA-LFQ LC–MS/MS) analysis

Peptide mixtures were analysed by liquid chromatography coupled to tandem mass spectrometry (LC–MS/MS) on an Orbitrap Astral mass spectrometer interfaced with a Vanquish Neo ultrahigh-performance liquid chromatography (UHPLC) system (Thermo Fisher Scientific). Peptides were first trapped on a PepMap trap cartridge (5 µm, 300 µm × 5 mm; Thermo Fisher Scientific) and separated on an EASY-Spray PepMap RSLC C18 analytical column (2 µm, 50 µm × 15 cm) maintained at 50 °C. The mobile phases consisted of buffer A (0.1% formic acid in water) and buffer B (0.1% formic acid in 80% acetonitrile), with a flow rate of 300 nl/min. The 15-min gradient was programmed as follows: 4–10% B for 2 min, 10–40% B for 11 min, 40–99% B for 0.5 min, and 99% B for 1.5 min.

Before injection, peptide samples were resuspended in 10 µl of buffer A, and 1 µl was injected for each run. Ionization was performed using nano-electrospray with a spray voltage of 1900 V and a capillary temperature of 280 °C. Data were acquired in data-independent acquisition (DIA) mode. Full MS scans covered the *m*/*z* range of 380–980 with a resolution of 240,000 (at *m*/*z* 200), an automatic gain control (AGC) target of 500%, and a maximum injection time (IT) of 5 ms. MS/MS spectra were acquired in the Astral analyser using an AGC target of 500%, an IT of 3 ms, and normalized collision energy (NCE) of 25. The scan range was set to *m*/*z* 380–980, with 2 *m*/*z* isolation windows and optimized window placement, resulting in 299 windows per cycle.

Raw DIA data were processed using Spectronaut v19.1 (Biognosys AG) with the directDIA workflow. Spectral library generation employed the UniProt human proteome database (UP000005640_9606.fasta, March 2024; 20,418 entries) and a dataset of common contaminants. The *D. immitis* proteome database retrieved from WormBase ParaSite (release WBPS19, BioProject PRJEB1797; 12,609 entries) was used due to the absence of a publicly annotated proteome for *D. repens* as the closest taxonomic reference for protein identification. Trypsin/P and LysC/P were defined as proteases, allowing up to two missed cleavages. Carbamidomethylation of cysteine was set as a fixed modification, while methionine oxidation and N-terminal acetylation were defined as variable. The false discovery rate (FDR) was controlled at 0.01 (0.1%) at the peptide–spectrum match (PSM), peptide, and protein levels. The IDPicker algorithm was used for protein inference, and automatic cross-run normalization was applied, while missing values were addressed through a global imputing strategy. Statistical analysis for differential abundance was performed using an unpaired Student *t*-test. Differentially regulated candidates were selected based on an absolute average (AVG) log2 fold change ≥ 0.58 and a *Q*-value ≤ 0.05.

### Bioinformatics analysis

Differential expression analysis was performed using Spectronaut software, establishing strict thresholds of absolute AVG log2 ratio ≥ 0.58, which corresponds to a ≥ 1.5-fold change, and a *Q*-value ≤ 0.05 for the determination of deregulated proteins. This fold change threshold in protein abundance was selected to ensure biologically relevant regulation of moderate magnitude, while statistical significance was ensured by the *Q*-value. The graphical representation of these findings included principal component analysis (PCA), which was calculated using the prcomp function from the stats package in R and visualized with ggplot22 [24] and volcano plots, for which differential expression metrics (log2 fold change and *Q*-values) were calculated using Spectronaut, and results were imported into R and visualized using ggplot2.

The functional characterization of differentially expressed proteins (DEPs) was performed using the STRING (Search Tool for the Retrieval of Interacting Genes/Proteins) database v.12 platform [25]. This analysis focused on Gene Ontology (GO) annotations, specifically in the biological processes category. The results of the enrichment analyses were visualized using the R ggplot2 package.

The identification of proteins associated with angiogenesis was performed using the GO database in the biological processes category of UniProt. Finally, both Venn diagrams, performed using the web tool InteractiVenn [26], and UpSet plot graphs, generated in R using the UpSetR package [27], were used to visualize and describe the expression patterns of the proteins.

## Results

### Effect of the treatments on cell viability and cytotoxicity

The viability and cytotoxicity of HUVECs exposed to VEGF-A, DrSA, Dr20/22, DrSA+VEGF-A, and Dr20/22+VEGF-A for 24 h remained comparable to those of untreated cultures. The proportion of viable cells exceeded 82.5% across all experimental conditions, with no significant differences observed between treated and non-stimulated cells (data not shown). Similarly, no cytotoxic effects were detected, as indicated by the lack of significant differences in adenylate kinase release between groups.

### Principal component analysis and volcano plots

Applying a confidence threshold of *Q* ≤ 0.01, a total of 572 protein groups were identified in the supernatants and 4432 in the cell lysates across all replicates (Supplementary Tables 1–6). Following the removal of common contaminants and proteins belonging to the *D. immitis* proteome, the lists were refined to 466 and 4373 protein groups for supernatants and lysates, respectively.

**Fig. 1:**
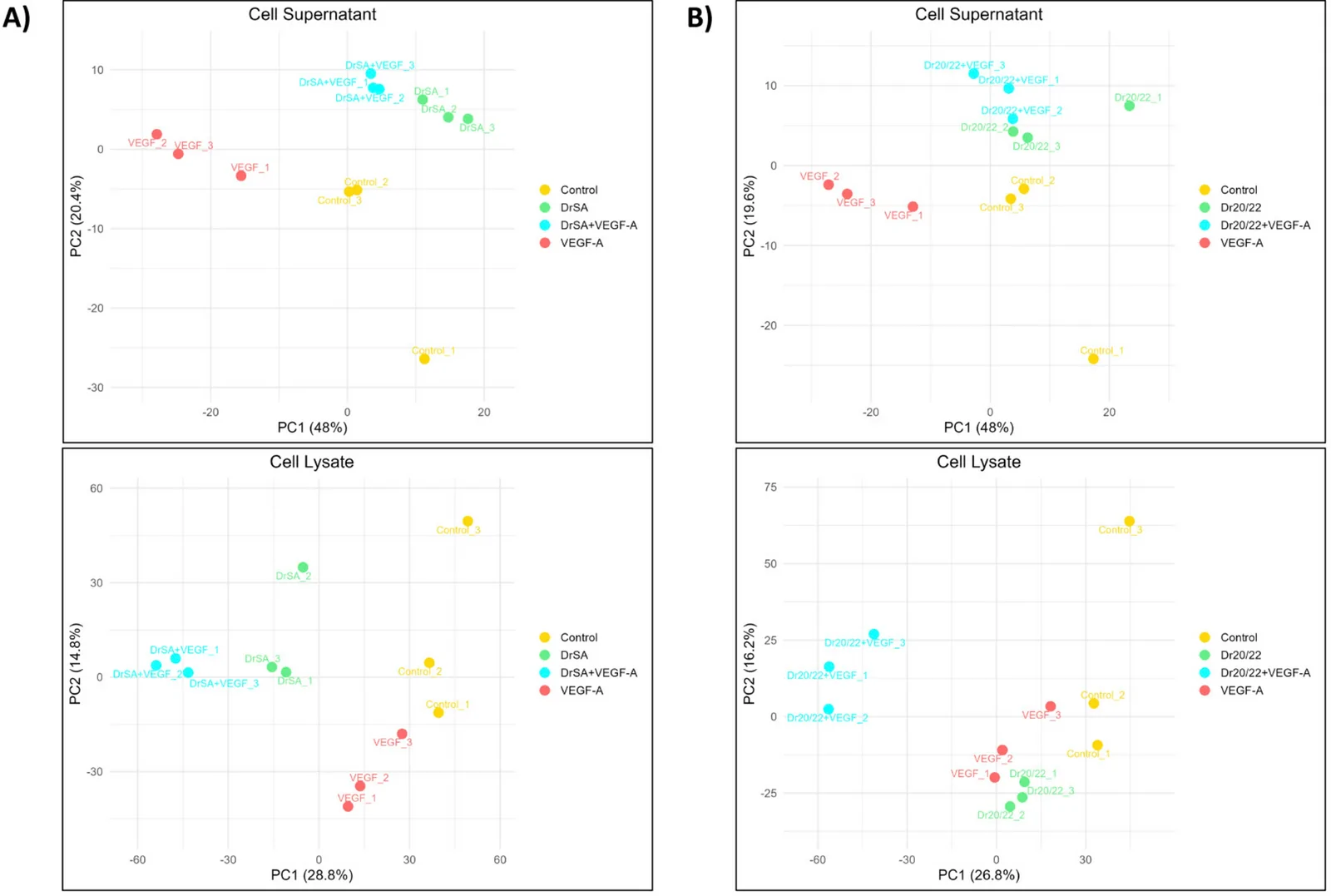
Principal component analysis (PCA) of proteins identified after stimulation with DrSA and DrSA+VEGF-A and control group (**A**) and Dr20/22, Dr20/22+VEGF-A, and VEGF-A and the control group (**B**) in cell supernatants and lysates

PCA was performed to visualize variations in protein expression profiles induced by stimulation (DrSA, DrSA+VEGF-A, VEGF-A) and the control group (Fig. 1). In the supernatant analysis, four distinct clusters were identified. The first principal component (PC1) accounted for 48% of the total variance, while the second (PC2) represented 20.4%. Similarly, PCA of cell lysates showed four clusters, with PC1 and PC2 explaining 28.8% and 14.8% of the variability, respectively. This pattern of four clusters was maintained in the groups involving Dr20/22, Dr20/22+VEGF-A, and VEGF-A stimulation and the control group. In this case, analysis of the supernatants showed greater variability (PC1 = 48%; PC2 = 19.6%). Cell lysate samples were also grouped into four clusters, although the explained variance was lower (PC1 = 26.8%; PC2 = 16.2%). In both groups, the highest proportion of variability corresponded to the proteins identified in the supernatant.

DEPs were identified using an absolute AVG log2 ratio ≥ 0.58 (AVG log2 ratio ≥ 0.58 for upregulated proteins and ≤ −0.58 for downregulated proteins) and a *Q*-value ≤ 0.05. In the supernatant (Supplementary Figure 1), VEGF-A stimulation caused the most significant alteration, with 269 DEPs (19 upregulated; 250 downregulated). DrSA stimulation deregulated 106 proteins (86 upregulated; 20 downregulated), while Dr20/22 altered 63 proteins (54 upregulated; nine downregulated). Among the co-stimulations, Dr20/22+VEGF-A induced the greatest shift, with 102 DEPs, compared to 88 (60 upregulated; 28 downregulated) for DrSA+VEGF-A.

Regarding cell lysates (Supplementary Figure 2), stimulation with Dr20/22+VEGF-A had the greatest impact, deregulating the levels of 483 proteins (327 upregulated; 156 downregulated), followed by stimulation with DrSA+VEGF, with 421 proteins (262 upregulated; 159 downregulated). Single stimulation with the antigens had a smaller impact in relation to the number of DEPs, with 210 proteins (137 upregulated; 73 downregulated) after stimulation with Dr20/22 and 268 (194 upregulated; 74 downregulated) after stimulation with DrSA. Stimulation with VEGF-A, on the other hand, caused the deregulation of 198 proteins (145 upregulated; 53 downregulated).

### Functional annotation

**Fig. 2:**
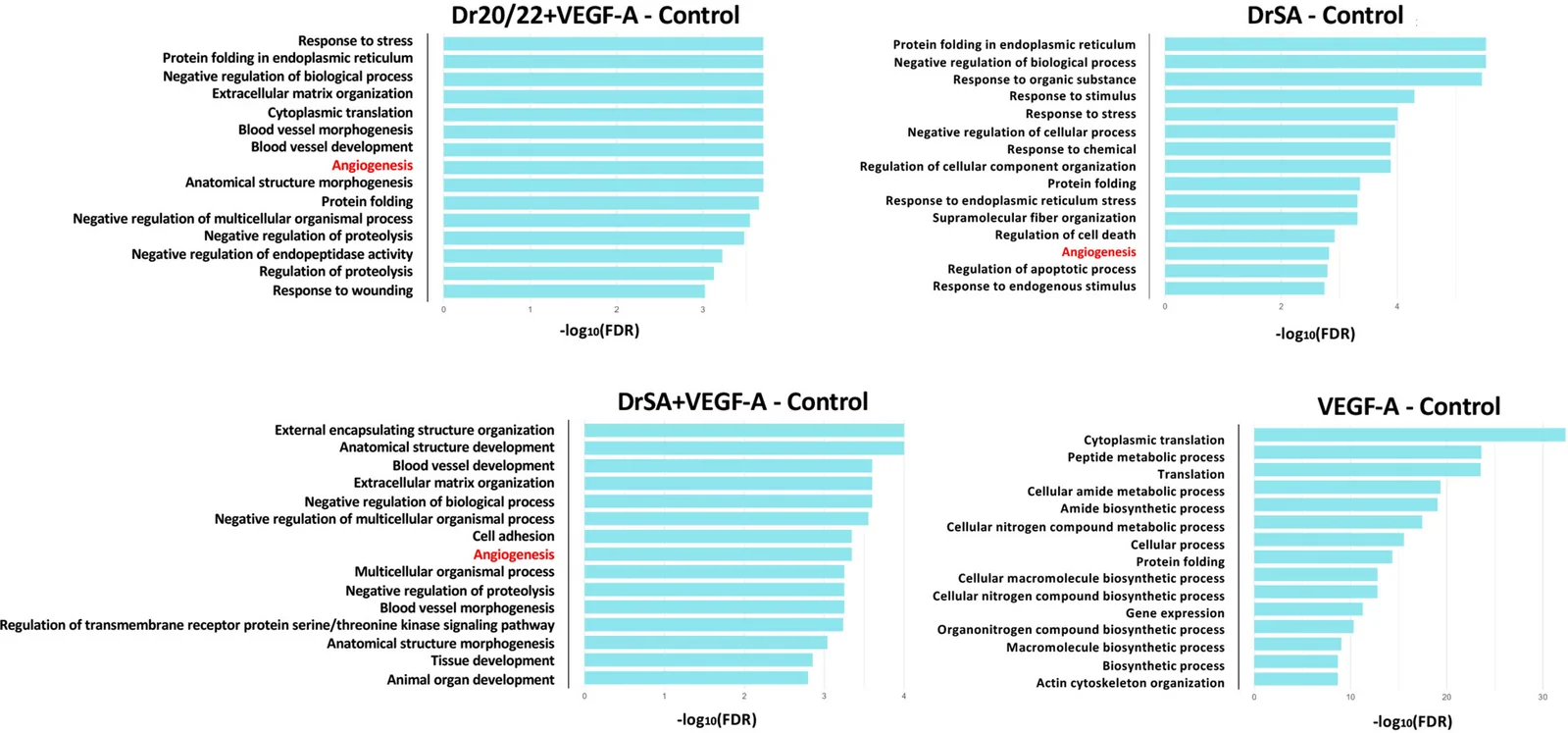
Gene Ontology (GO) enrichment analysis in biological process category of differentially expressed proteins in cell supernatants

Functional interpretation of protein deregulation was conducted through GO analysis focusing on biological processes via the STRING database (Fig. 2). GO analysis was applied to the lists of proteins that showed deregulation when comparing each of the conditions with the control group.

Functional analysis was restricted to proteins identified in the cell supernatant, as secreted or released proteins more accurately reflect the cellular response to stimulation than cell lysates, in which the high abundance of proteins could mask specific biological processes that were altered after treatment.

In the DrSA+VEGF-A group, angiogenesis was identified as the eighth most enriched biological process (FDR = 0.00045), whereas enrichment was lower for DrSA alone (FDR = 0.0015). For Dr20/22+VEGF-A, angiogenesis also ranked eighth (FDR = 2.01 × 10^−6^); however, this process did not reach significance following stimulation with Dr20/22 alone. Notably, angiogenesis was not among the primary enriched processes in the VEGF-A-only stimulation.

### Identification of proteins related to angiogenesis

**Fig. 3:**
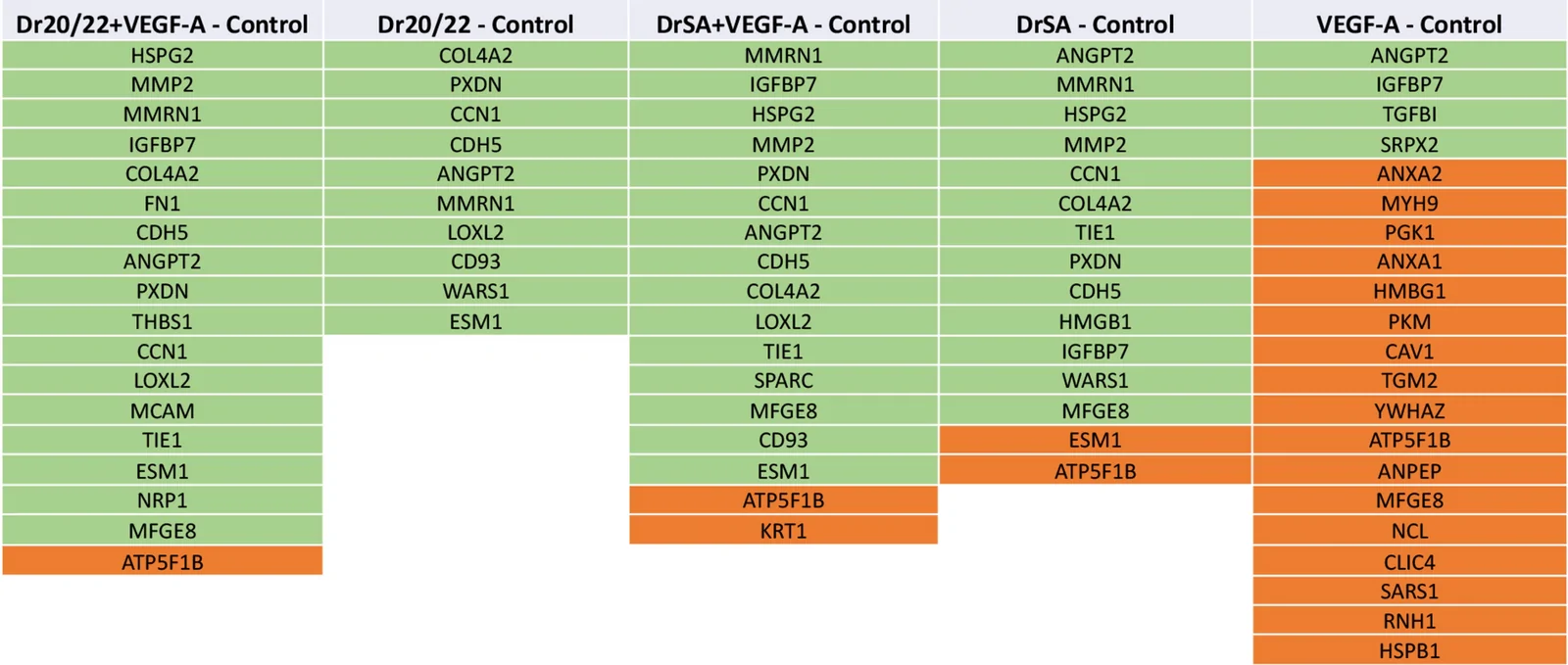
Upregulated () and downregulated () angiogenesis proteins after stimulation compared to the control group in cell supernatants

To isolate DEPs specifically involved in angiogenesis, UniProt GO annotations were filtered. In the supernatant (Fig. 3), co-stimulation with antigens and VEGF-A induced similar responses: 17 DEPs (15 upregulated; two downregulated) for DrSA+VEGF-A and 18 (17 upregulated; one downregulated) for Dr20/22+VEGF-A, predominantly characterized by upregulation. In contrast, VEGF-A stimulation primarily induced downregulation (17 out of 21 proteins). On the other hand, single stimulation with DrSA produced a greater response by deregulating the levels of 16 proteins (14 upregulated; two downregulated) than Dr20/22, which deregulated 10 proteins, all of which were overexpressed.

**Fig. 4:**
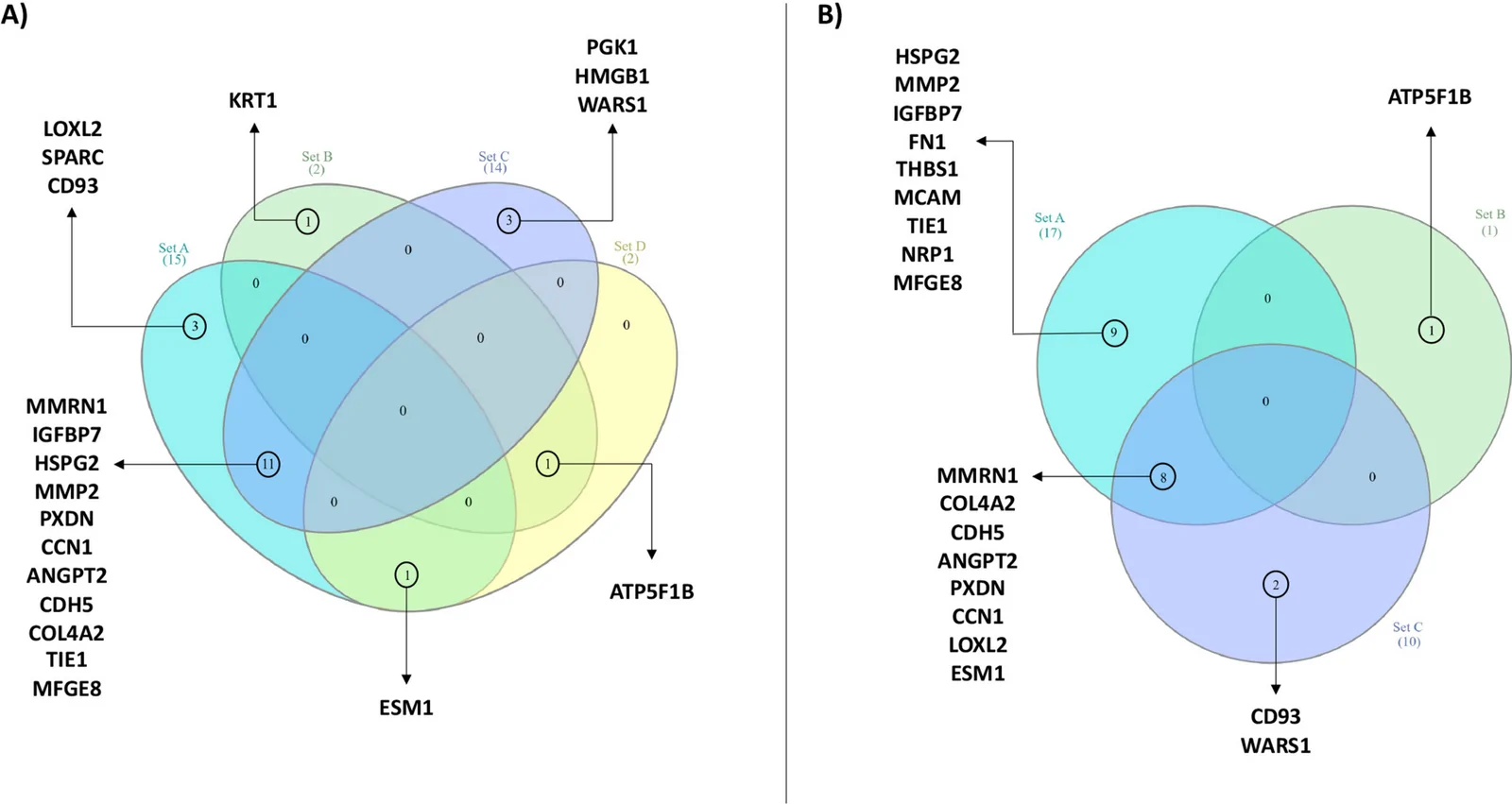
Venn diagram of differentially expressed proteins after all treatments in cell supernatant. **A** Differential expression of proteins between treatments with DrSA+VEGF-A (Set A upregulated; Set B downregulated) and DrSA (Set C upregulated; Set D downregulated). **B** Differential expression of proteins between treatments with Dr20/22+VEGF-A (Set A upregulated; Set B downregulated) and Dr20/22 (Set C upregulated)

Angiopoietin-2 (ANGPT2) was upregulated across all treatments, alongside cadherin-5 (CDH5), multimerin-1 (MMRN1), and CCN family member 1 (CCN1). Other factors, such as endothelial cell-specific molecule 1 (ESM1) and matrix metalloproteinase-2 (MMP2), were increased except for DrSA (where ESM1 decreased) and Dr20/22 (where MMP2 remained unchanged). Notably, tryptophanyl-tRNA synthetase 1 (WARS1) was elevated exclusively after treatment with DrSA and Dr20/22 (Fig. 4).

**Fig. 5:**
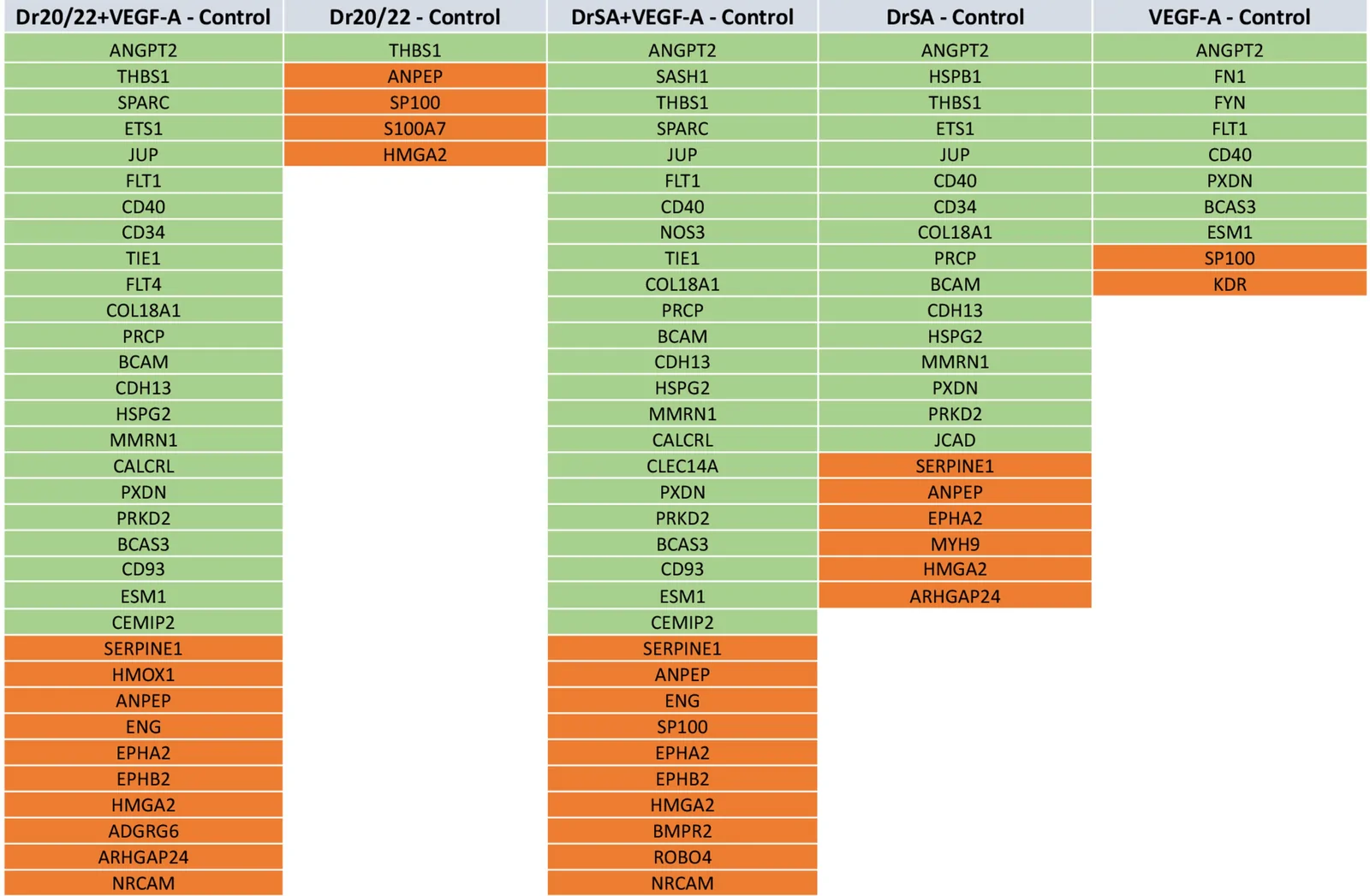
Upregulated () and downregulated () angiogenesis proteins after stimulation compared to the control group in cell lysates

Analysis of cell lysates showed that antigen stimulations supplemented with VEGF-A had the greatest impact on protein regulation (Fig. 5), altering the levels of 33 proteins (23 upregulated; 10 downregulated) in both treatments. Single stimulation with DrSA modulated 22 proteins (16 upregulated; six downregulated), while stimulation with Dr20/22 had a basal effect, altering only five proteins (one upregulated; four downregulated). In addition, treatment with VEGF-A deregulated 10 proteins (eight upregulated; two downregulated).

**Fig. 6:**
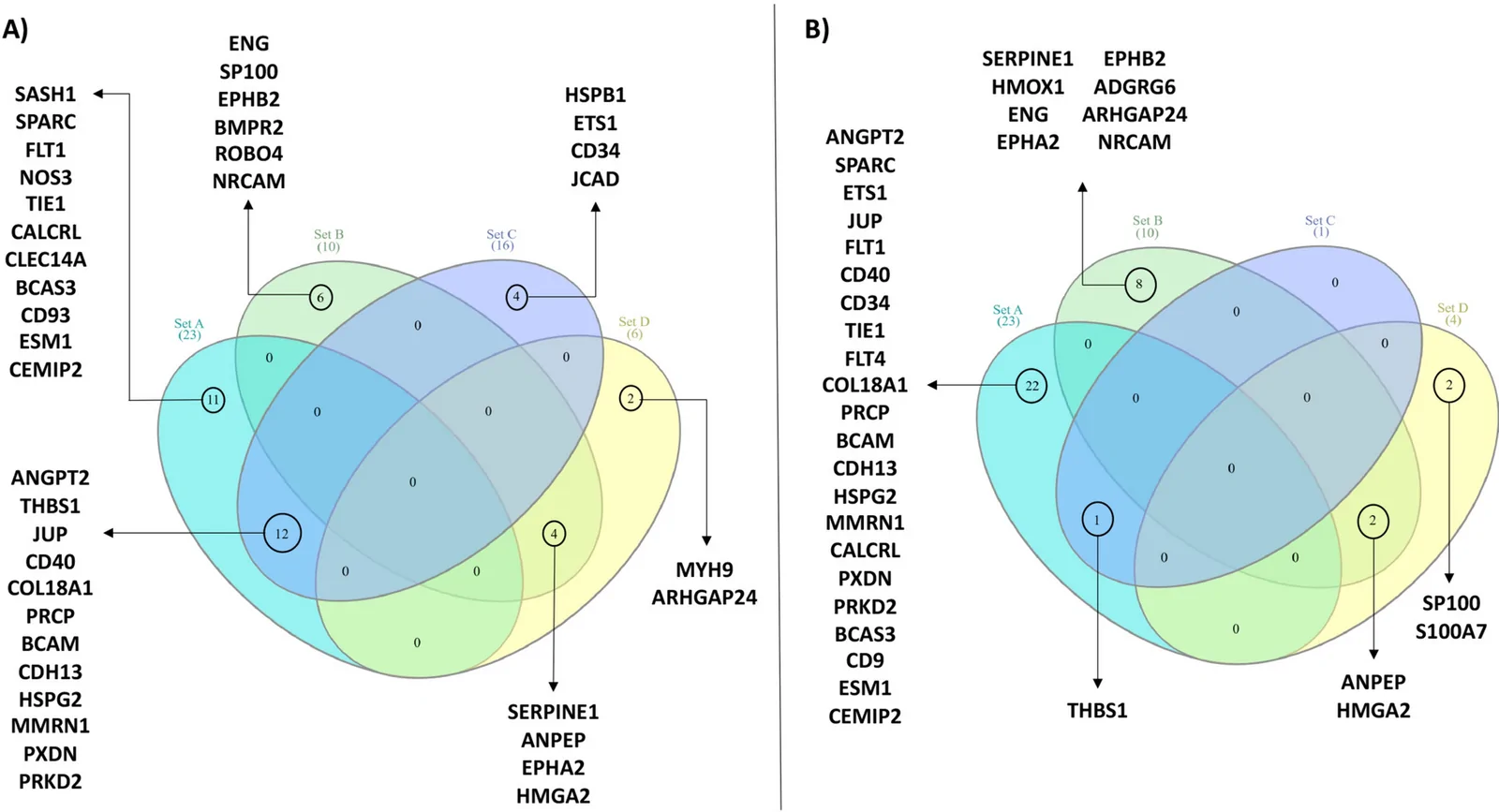
Venn diagram of differentially expressed proteins after all treatments in cell lysates. **A** Differential expression of proteins between treatments with DrSA+VEGF-A (Set A upregulated; Set B downregulated) and DrSA (Set C upregulated; Set D downregulated). **B** Differential expression of proteins between treatments with Dr20/22+VEGF-A (Set A upregulated; Set B downregulated) and Dr20/22 (Set C upregulated; Set D downregulated)

At the intracellular level, ANGPT2 remained elevated in all conditions except for stimulation with Dr20/22. Additionally, the groups supplemented with VEGF-A showed upregulation of the vascular endothelial growth factor receptor 1 (FLT1 or VEGFR1) and tyrosine kinase receptor 1 (TIE1), as well as the ESM1 protein. Relevantly, only treatment with DrSA+VEGF-A exhibited an expression profile characterized by upregulation of NOS3 and downregulation of Roundabout guidance receptor 4 (ROBO4) (Fig. 6).

**Fig. 7:**
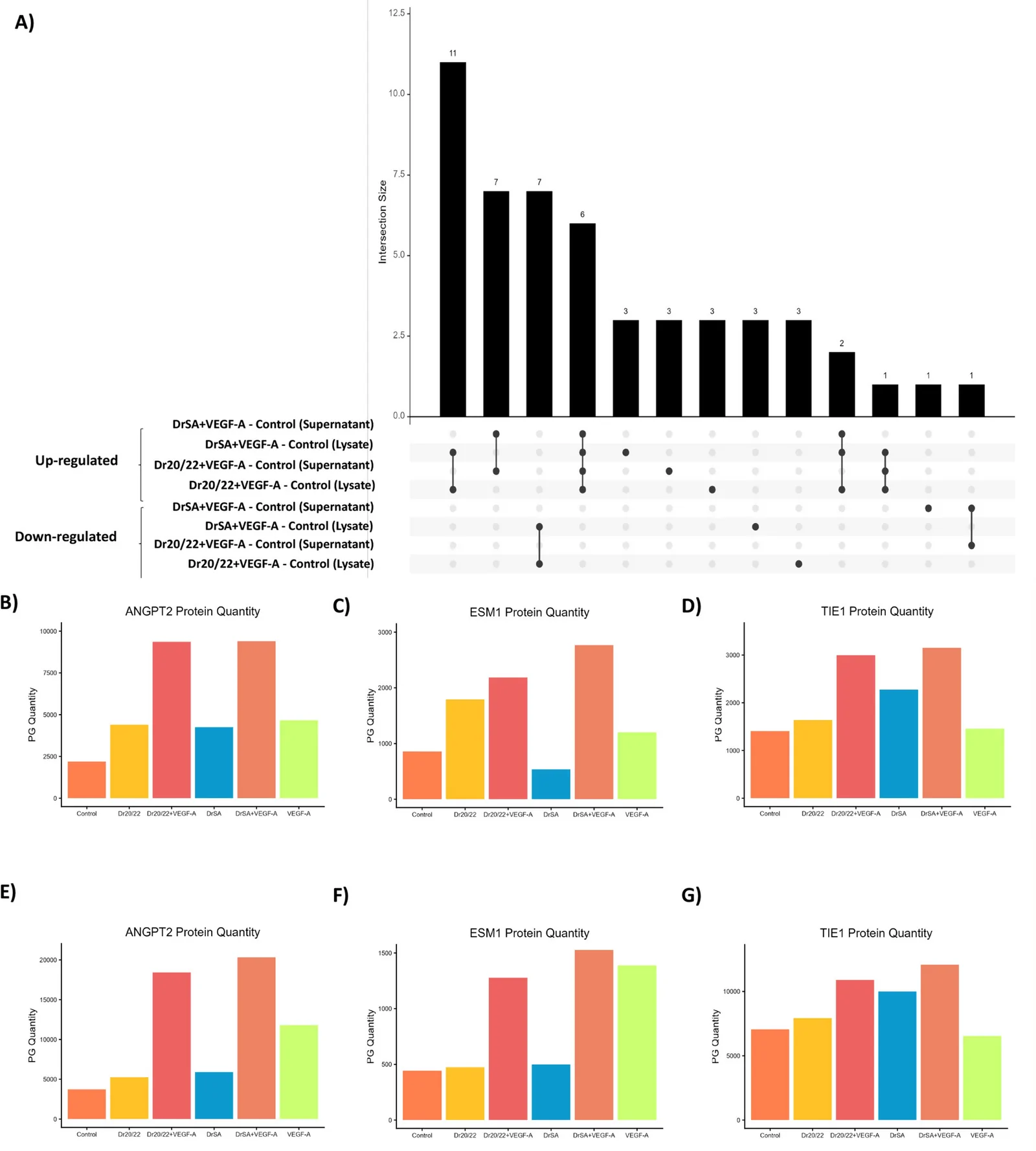
**A** UpSet plot of differentially expressed proteins after stimulation with antigens supplemented with VEGF-A in cell supernatants and lysates. Differentially expressed proteins after treatments with antigen supplemented with VEGF-A in cell supernatants (**B–D**) and cell lysates (**E–G**)

Finally, it was confirmed that after stimulation with DrSA+VEGF-A and Dr20/22+VEGF-A, a total of six common proteins were identified as overexpressed in both compartments (supernatant vs. lysate) (Fig. 7), among which ANGPT2, TIE1, and ESM1 stand out. Additionally, seven upregulated proteins were common in supernatant and lysate in stimulation with DrSA+VEGF-A, among which MMP2 stands out, and 11 proteins after stimulation with Dr20/22+VEGF-A, among which FLT1 stands out.

## Discussion

Under physiological conditions, endothelial cells remain in a state of quiescence; however, when faced with pathological stimuli, they are activated to initiate angiogenesis, a coordinated process of cell proliferation, migration, and differentiation mediated by pro-angiogenic factors such as VEGF-A [28–30]. Previous studies have indicated that DrSA, supplemented with VEGF-A, enhances this process by increasing the expression of VEGFR2 and endoglin, facilitating the formation of pseudocapillary structures in vitro [20].

The present study delves deeper into these findings through endothelial proteomic characterization, evaluating not only the effect of DrSA on the angiogenic process but also the effect of Dr20/22, a larval-specific antigen associated with early host–parasite interaction. Although the biological function of Dr20/22 remains unknown, its association with early tissue invasion suggests a potential role in modulating endothelial or immune cell responses [16, 21, 22].

In the cell supernatant, stimulation with VEGF-A induced the greatest deregulation of angiogenic proteins; however, most were downregulated. Among these, intracellular or membrane proteins were identified, such as annexins or MYH9, a profile that could be more strongly associated with the cytoprotective effect exerted by VEGF than with direct stimulation of angiogenesis [31]. In contrast, protein overexpression predominated in the other treatments, with no alterations in the levels of classically intracellular proteins. In quantitative terms, both the treatments with VEGF-A-supplemented antigens and the single treatment with DrSA showed similar effects on the number of dysregulated proteins, unlike the Dr20/22 protein, whose impact was lower. These data are consistent with GO analyses, where angiogenesis emerged as a significantly enriched biological process only after stimulation with antigens supplemented with VEGF-A and DrSA, while with VEGF-A or Dr20/22 it did not reach major enrichment levels.

A cross-sectional increase in ANGPT2 levels was observed in both lysates and supernatants after treatment (except in the Dr20/22 lysate), indicating an increase in its synthesis and secretion. This finding correlates with a modulation of the ANGPT1/TIE2 axis, responsible for vascular stability [32]. The function of ANGPT2 is context-dependent: in the presence of VEGF-A, it acts in a pro-angiogenic manner by promoting the vascular destabilization necessary for proliferation; however, in the absence of VEGF-A, elevated levels of ANGPT2 are associated with a vascular regression phenotype [33, 34]. This suggests that only stimulation with VEGF-A-supplemented antigens induces a true angiogenic phenotype, while stimulation with antigens alone may promote regression. Additionally, co-stimulation with VEGF-A maintained overexpression of the proteins TIE1 and ESM1, the latter recognized as a biomarker of pro-angiogenic endothelial dysfunction [35]. It is noteworthy that ESM1 was downregulated in the supernatant after single treatment with DrSA, suggesting reduced secretion in the absence of external growth factors.

Cell lysates revealed that the synergy between parasitic antigens and VEGF-A has the greatest impact on the angiogenic response. While Dr20/22 and VEGF-A alone barely modulated specific proteins (5 and 10 proteins, respectively), the combination of antigens with VEGF-A significantly enhanced the response, as has been seen in previous research [19, 20]. Specifically, treatment with DrSA+VEGF-A induced the overexpression of NOS3, a regulator of endothelial function and nitric oxide production [36], which is essential for VEGF-induced vascular permeability [37, 38]. Simultaneously, a decrease in ROBO4, a receptor that normally inhibits cell migration and proliferation, was observed. The reduction in ROBO4, coupled with the increase in NOS3, suggests a molecular environment that favours endothelial hyperpermeability and the angiogenic progression characteristic of *D. repens* pathology [39, 40].

## Conclusions

Our findings confirm previous research on the ability of *D. repens* to modulate the angiogenic process in pro-angiogenic environments stimulated with VEGF-A. In this context, the central role of the ANGPT2 protein stands out, whose dual role is decisive in directing the endothelial response toward a phenotype of active remodelling or vascular regression, depending on the availability of growth factors. Likewise, the specific alteration in NOS3 and ROBO4 levels observed exclusively after stimulation with DrSA+VEGF-A represents an unprecedented finding that expands our knowledge of the mechanisms of hyperpermeability and vascular activation induced by the parasite. These results underscore the importance of conducting additional studies to further characterize these molecular pathways during host–parasite interaction in order to elucidate the etiopathogenesis of lesions.

## Supplementary Information

Supplementary Material 1. Figure 1. Differential expression of proteins in cell supernatant according to an absolute AVG log2 ratio ≥ 0.58 and a *Q*-value ≤ 0.05 after stimulation with the different treatments compared to the control group

Supplementary Material 2. Figure 2. Differential expression of proteins in cell lysates according to an absolute AVG log2 ratio ≥ 0.58 and a *Q*-value ≤ 0.05 after stimulation with the different treatments compared to the control group

Supplementary Material 3. Table 1. Protein quantity of identified proteins after all treatments and control group in cell supernatants

Supplementary Material 4. Table 2. Protein quantity of identified proteins after all treatments and control group in cell lysates

Supplementary Material 5. Table 3. Differential expression of proteins identified in the supernatants between the different groups

Supplementary Material 6. Table 4. Differential expression of proteins identified in the lysates between the different groups

Supplementary Material 7. Table 5. Differentially up- and downregulated proteins in cell supernatants with a *Q*-value ≤ 0.05 and absolute AVG log2 ratio ≥ 0.58 between different groups

Supplementary Material 8. Table 5. Differentially up- and downregulated proteins in cell lysates with a *Q*-value ≤ 0.05 and absolute AVG log2 ratio ≥ 0.58 between different groups

## Data Availability

The datasets supporting the conclusions of this article are included within the article.

## Abbreviations

ANGPT2: Angiopoietin-2
AGC: Automatic gain control
CDH5: Cadherin-5
CCN1: CCN family member 1
*D. immitis*: *Dirofilaria immitis*
*D. repens*: *Dirofilaria repens*
DC: Detergent-compatible
ESM1: Endothelial cell-specific molecule 1
EDTA: Ethylenediaminetetraacetic acid
FDR: False discovery rate
FGF: Fibroblast growth factors
GO: Gene Ontology
HUVEC: Human umbilical vein endothelial cell
IT: Injection time
LC: Liquid chromatography
MS/MS: Mass spectrometry
MMP2: Matrix metalloproteinase-2
MAPK: Mitogen-activated protein kinase
MMRN1: Multimerin-1
NCE: Normalized collision energy
PBS: Phosphate-buffered saline
PI3K: Phosphatidylinositol-3 kinase
PCR: Polymerase chain reaction
PCA: Principal component analysis
AKT or PKB: Protein kinase B
ROBO4: Roundabout guidance receptor 4
DrSA: *D. repens* somatic antigens
L_3_: Third-stage larvae
TCEP: Tris(2-carboxyethyl)phosphine
WARS1: Tryptophanyl-tRNA synthetase 1
TIE: Tyrosine kinase receptor
VEGF: Vascular endothelial growth factor
FLT1 or VEGFR1: Vascular endothelial growth factor receptor 1
VEGFR2: Vascular endothelial growth factor receptor 2
